# LINC01088/miR-22/CDC6 Axis Regulates Prostate Cancer Progression by Activating the PI3K/AKT Pathway

**DOI:** 10.1155/2023/9207148

**Published:** 2023-07-19

**Authors:** Jianwei Li, Xinghua Huang, Haodong Chen, Caifu Gu, Binyu Ni, Jianhua Zhou

**Affiliations:** ^1^Department of Urology, Longgang District People's Hospital of Shenzhen, Guangdong 518000, China; ^2^Department of Thyroid and Breast Surgery, Longgang Central Hospital, Shenzhen, Guangdong 518000, China; ^3^Department of Pediatrics, Longgang District People's Hospital of Shenzhen, Shenzhen, Guangdong 518000, China

## Abstract

**Background:**

Prostate cancer (PCa) harms the male reproductive system, and lncRNA may play an important role in it. Here, we report that the LINC01088/microRNA- (miRNA/miR-) 22/cell division cycle 6 (CDC6) axis regulated through the phosphatidylinositide 3-kinases- (PI3K-) protein kinase B (AKT) signaling pathway controls the development of PCa.

**Methods:**

lncRNA/miRNA/mRNA associated with PCa was downloaded and analyzed by Gene Expression Omnibus. The expression and correlation of LINC01088/miR-22/CDC6 in PCa were analyzed and verified by RT-qPCR. Dual-luciferase was used to analyze the binding between miR-22 and LINC01088 or CDC6. Cell Counting Kit-8 and Transwell were used to analyze the effects of LINC01088/miR-22/CDC6 interactions on PCa cell viability or migration/invasion ability. Localization of LINC01088 in cells was analyzed by nuclear cytoplasmic separation. The effect of LINC01088/miR-22/CDC6 interaction on downstream PI3K/AKT signaling was analyzed by Western blot.

**Results:**

LINC01088 or CDC6 was upregulated in prostate tumor tissues or cells, whereas miR-22 was downregulated, miR-22 directly targets both LINC01088 and CDC6. si-LINC01088 inhibits the PCa process by suppressing the PI3K/AKT pathway. CDC6 reverses si-linc01088-mediated cell growth inhibition and reduction of PI3K and AKT protein levels.

**Conclusion:**

Our results demonstrate that the LINC01088/miR-22/CDC6 axis functions in PCa progression and provide a promising diagnostic and therapeutic target.

## 1. Introduction

Prostate cancer (PCa) is a cancer that affects men and is very common worldwide. It accounts for 21% of all new cases of cancer in men. Unfortunately, it causes significant illness and death in those affected [1, 2]. Early systematic screening has a very positive effect on the prevention and treatment of prostate cancer, and the available set of PC biomarkers is growing [3]; there is still a significant unmet need for new and more effective treatments.

Long noncoding RNAs (lncRNAs), which are greater than 200 nucleotides and do not code for proteins, significantly impact cancer tumorigenesis and metastasis when their expression is altered [4]. lncRNAs primarily function through the regulation of competing endogenous RNAs (ceRNAs), which act as sponges for miRNAs that would otherwise target specific genes. Several lncRNAs, including MEG3, FOXP4-AS1, and H19, have been implicated in prostate cancer [5–7]. However, the molecular mechanism by which LINC01088, which is considered an oncogene in most cancers [8, 9], operates in prostate cancer has not yet been elucidated.

miR-22 impedes the progression of different cancer types, including bladder, colorectal, and gastric cancers [10–12]. Similarly, miR-22, which is an androgen receptor (AR) cistrome member, suppresses process of cancer cells such as LNCaP or PC3 in PCa [13]. The ceRNA role of miRNA targeting lncRNAs or mRNA 3′untranslated region (UTR) in PCa has been reported. For instance, miR-22 targets the HOTAIR and 3′UTR of *HMGB1*, affecting PCa prognosis [14]. Additionally, miR-22 has been shown to regulate multiple signaling pathways in PCa cells. Specifically, miR-22 activates Wnt/*β*-catenin signaling in carcinoma [15]. Dhar et al. found that MTA1-activated miR-22 regulates PCa invasiveness [16]. Moreover, miR-22 regulates the PI3K/AKT pathway in various cancers, such as ovarian cancer and osteosarcoma [17, 18]. Nevertheless, whether miR-22 activates the PI3K/AKT pathway in PCa remains unclear.

Cell division cycle 6 (CDC6) is a novel cancer target that regulates the DNA replication process and is considered an early indicator of malignancy [19]. CDC6 has been recognized as a cancer diagnosis biomarker in various cancers [20, 21]. For instance, Mahadevappa et al. found the prognostic significance of CDC6 in breast cancer [22]. However, only few studies have investigated the CDC6 expression in PCa [23]; therefore, CDC6 requires further investigation.

The AR and frequent activation of PI3K signaling are key factors in the progression of PCa [24]. The PI3K/AKT signaling pathway, which is cross-regulated through several reciprocal inhibitory loops with AR pathways, is the driver of prostate tumor development [25, 26]. Recent research by Tan et al. showed that PI3K/AKT axis inactivation suppresses prostate tumorigenesis [27]. Dai et al. confirm that inhibition of the PI3K/AKT signaling pathway facilitates PCa cell autophagy [28]. PI3K and AKT inhibitors will soon be introduced as antitumor drugs and are currently in preclinical and clinical development [29–31].

This study is aimed at understanding the interaction and mechanism of the LINC01088/miR-22/CDC6 axis and exploring its possibility as a biomarker for systematic screening of PCa.

## 2. Materials and Methods

### 2.1. Samples

Ten pairs of fresh frozen PCa and adjacent tissues were obtained from the Longgang District People's Hospital of Shenzhen (Shenzhen, China), along with pathological information. All patients were histologically confirmed to have a prostate tumor. The experiments was approved by the institutional ethics committees of Longgang District People's Hospital of Shenzhen (Shenzhen, China; approval no. 2022071) and conducted in accordance with the Declaration of Helsinki.

### 2.2. Microarray Raw Data Analyses

Datasets were from Gene Expression Omnibus. The series (GSE104749) dataset were utilized to analyze lncRNA/mRNA expression profiles in PCa and categorized as follows: control (4 benign prostatic hyperplasia (BPH) fine-needle aspiration biopsy tissues), and PCa (4 PCa fine-needle aspiration biopsy tissues). The GEO series (GSE45604) dataset was utilized to analyze miRNA expression profiles in PCa and categorized as follows: normal (10 normal prostate tissues) and PCa (10 PCa tissues). Expression of dysregulated RNA was identified to meet the |fold change| ≥ 1.5 criteria. GEPIA2 was used for The Cancer Genome Atlas (TCGA) survival analysis. Candidate genes and cancer subtypes were chosen as the inputs to generate survival curves for disease-free survival. Statistical significance was defined as *P* < 0.05.

### 2.3. Predicted Targets of LINC01088 and miR-22

The interactions between LINC01088 and miR-22 were predicted using LncBase v.3 (https://diana.e-ce.uth.gr/lncbasev3/interactions). Putative targets of miR-22 were predicted using TargetScan (http://www.targetscan.org/vert_72/) and TarBase v.8 (https://dianalab.e-ce.uth.gr/html/diana/web/index.php?r=tarbasev8%2Findex).

### 2.4. Cell Culture and Transfection

Human PCa cell lines (LNCAP, PC3, C4-2, and 22RV1) and prostate epithelial cells (RWPE-2) were purchased from American Type Culture Collection (ATCC; VA, USA). LNCAP, PC3, C4-2, 22RV1, and RWPE-2 cells were maintained in RPMI-1640 (HyClone, UT, USA) with 10% fetal bovine serum (FBS; HyClone). Lipofectamine 3000 (Invitrogen; Thermo Fisher Scientific, Inc.) were transfected into cells and used in subsequent experiments 48 h later.

### 2.5. Nucleocytoplasmic Separation and RT-qPCR Assay

Cytoplasmic and Nuclear RNA Purification Kit (bioWORLD; OH, USA) was isolated from cells according to the manufacturer's instructions. Total RNA was extracted from tissues or cells using a TRIzol reagent. After centrifugation at 12000 × g (4°C, 10 min), RNA was reverse-transcribed to cDNA and analysis using One-Step SYBR Green RT-qPCR Kit (Biomarker, Beijing, China) and performed at conditions: 95°C for 1 min followed by 40 cycles of 95°C for 6 s and 60°C for 32 s. Sequences of the primers were as follows: *LINC01088* forward, 5′-TAGGGTGCCTTCACCTGCTA-3′ and *LINC01088* reverse, 5′-TACACCCGGTGGAAAACTCC-3′; *CDC6* forward, 5′-GATCAACTGGACAGCAAAGG-3′ and *CDC6* reverse, 5′-CTAGGTAGAATTCTATCTGT-3′; miR-22 forward, 5′-ACACTCCAGCTGGGAGTTCTTCAGTGGCAA-3′ and miR-22 reverse, 5′-CTCAACTGGTGTCGTGGA-3′; 18sRNA forward, 5′-CCTGGATACCGCAGCTAGGA-3′ and 18sRNA reverse, 5′-GCGGCGCAATACGAATGCCCC-3′; and U6 forward, 5′-CTCGCTTCGGCAGCACA-3′ and U6 reverse, 5′-AACGCTTCACGAATTTGCGT-3′. RNA levels were calculated using the 2^−*ΔΔ*Ct^ method [32].

### 2.6. RNA Pull-Down Assay

Biotin-labeled *LINC01088* and miR-22 were transfected into PCa cells. After 24 h, cells were lysed with RIPA lysis buffer and then incubated (12 min) with Dynabeads M-280 Streptavidin (Invitrogen), followed by RT-qPCR analysis. Biotinylated RNA was obtained from Sangon Biotech.

### 2.7. Transient LINC01088 Silencing

The siRNA sequences were as follows: si-LINC01088-1, 5′-CCTTAAAGTAGCAATCTTAdTdT-3′; si-LINC01088-2, 5′-GAGAAATTGGACCAGACAAdTdT-3′; si-LINC01088-3, 5′-AGTCTGCATTGAAGATGTAdTdT; and si-NC, 5′-TTCTCCGAACGTGTCACGdTdT-3′.

### 2.8. Cell Viability Assay

To assess cell viability, PC3 and LNCAP cells (4 × 10^3^) were treated with 10 *μ*L of CCK-8 reagent (Solarbio) at 0, 24, 48, and 72 hours. After 60 min of incubation (in the dark) at 37°C, the absorbance of the samples was determined at 450 nm using an enzyme-labeled instrument (Thermo Fisher Scientific).

### 2.9. Migration and Invasion Assays

Migration of PC3 and LNCAP cells was verified using Transwell inserts (BD Biosciences) with a porous polycarbonate membrane. Cells were maintained in serum-free medium (upper chamber), and a medium (10% FBS) was added to the lower chamber, for 24 h. The difference with migration is that the invasion assay preincubates Matrigel (BD Biosciences) on Transwell inserts. Then, it was fixed with absolute alcohol, stained with crystal violet, and counted.

### 2.10. Dual-Luciferase Reporter Assay

Inserted *LINC01088* or *CDC6* 3′UTR into psi-CHECK2 and transfected into 293 T cells (ATCC) with miR-22 mimic using lipofectamine 3000 at 37°C for 4 h. Luciferase activity was measured using the dual-luciferase reporter assay system (Promega) at 490 nm after 48 h of culture. Firefly luciferase values were normalized using the ratio of firefly to Renilla luciferase activity.

### 2.11. Western Blotting

Denatured proteins from PCa cells were resolved by 10% SDS-PAGE (Beyotime). The separated bands were subsequently transferred to PVDF membranes and incubated (12 h; 4°C) with PI3K (1 : 1200, ab191606), AKT (1 : 500, ab8805), p-PI3K (1 : 500, ab182651), p-AKT (1 : 500, ab38449), CDC6 (1 : 1,000, ab109315), and GAPDH (1 : 3000, ab8245) primary antibodies. They were then incubated (2 h, 25°C) with goat anti-rabbit antibody (1 : 12,000, ab205718). Antibodies were obtained from Abcam (Cambridge, UK).

### 2.12. Statistical Analysis

Mean ± SD values were used to present the data, and GraphPad Prism 9 was utilized for statistical analysis. Initially, one-way ANOVA was performed, followed by a Bonferroni post hoc test to establish the presence of a statistically significant change overall. Subsequently, a Student's *t*-test was employed to examine the difference between any two groups. Statistical significance was defined as *P* < 0.05.

## 3. Results

### 3.1. LINC01088 Is Upregulated in PCa

**Figure 1:**
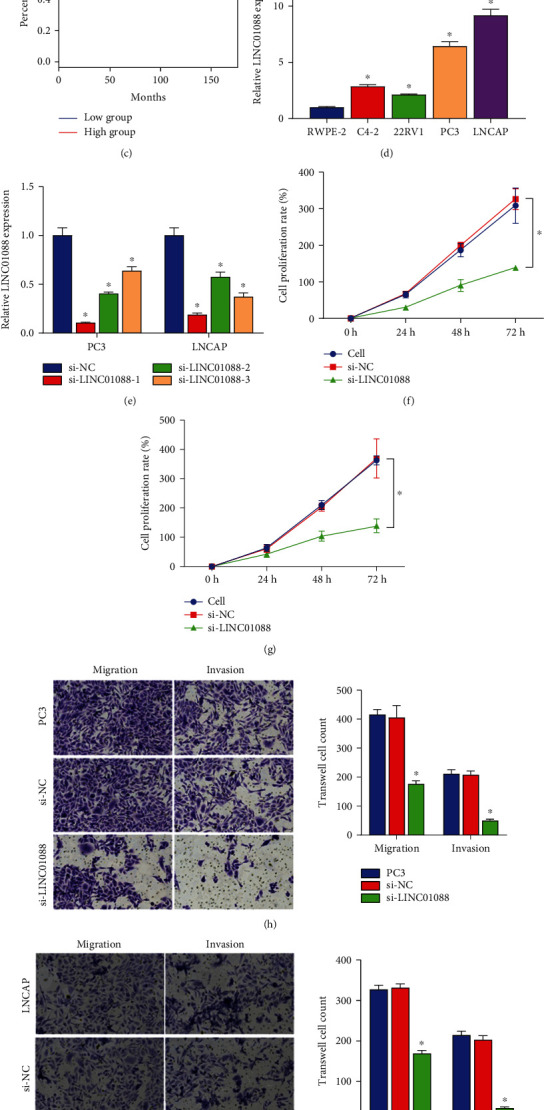
LINC01088 is a new potential target that promotes PCa development. (a) Hierarchical clustering heat map shows differentially expressed lncRNA including LINC01088 between the PCa and control groups. (b) Relative expression levels of LINC01088 between the PCa tumor tissues and adjacent tissues. (c) The disease-free survival rate was calculated in the LINC01088-high or LINC01088-low groups by TCGA survival analysis on the GEPIA2 database. (d) Relative expression of LINC01088 in RWPE-2 and four PCa cell lines (C4-2, 22RV1, PC3, and LNCaP). (e) RT-qPCR analysis of the inhibition efficiency of 3 si-LINC01088 in PC3 and LNCAP cells. CCK8 analysis of the effect of si-LINC01088 on the viability rate of (f) PC3 and (g) LNCAP cells. Transwell analysis of the effects of si-LINC01088 on migration and invasion of (h) PC3 and (i) LNCAP cells. (j) RT-qPCR analysis of LINC01088 localization in PC3 and LNCAP cells. ^∗^*P* < 0.05.

We conducted a comprehensive inquiry into the potential role of a recently discovered long noncoding RNA (lncRNA) in the advancement of prostate cancer (PCa). Our investigation primarily involved lncRNA chip data, which revealed that 45 differentially expressed transcripts lncRNAs, were identified in PCa specimens compared to the control group. Of these transcripts, LINC01088 exhibited the most significant log-fold change (logFC) value, thereby implying its potential as a target for further research (Figure 1(a)). Additionally, we validated the overexpression of LINC01088 in PCa tissues (Figure 1(b)), which was linked with decreased disease-free survival in PCa patients, and associated with diverse clinicopathological features of PCa (Figure 1(c)). The expression of LINC01088 was notably heightened in PCa cells in comparison to RWPE-2 cells (Figure 1(d)), particularly in PC3 and LNCAP cells, highlighting the significant role of LINC01088 in PCa progression. Subsequent findings illustrated that siRNA-LINC01088-1 was the most potent inhibitor of LINC01088 among the three siRNAs in PC3 and LNCAP cells, and we used it as an antagonist for further molecular mechanistic studies (Figure 1(e)). Our findings indicate that the reduction of LINC01088 expression results in a diminished cellular viability, as well as decreased migratory and invasive capacity of both PC3 and LNCAP cell lines (as depicted in Figures 1(f)–1(i)). Moreover, based on our observations, LINC01088 is preferentially localized in the cytoplasmic region of these cells (Figure 1(j)), implying that its effect on prostate cancer pathophysiology may be mediated by a competing endogenous RNA (ceRNA) mechanism.

### 3.2. LINC01088 Directly Targeted miR-22

**Figure 2:**
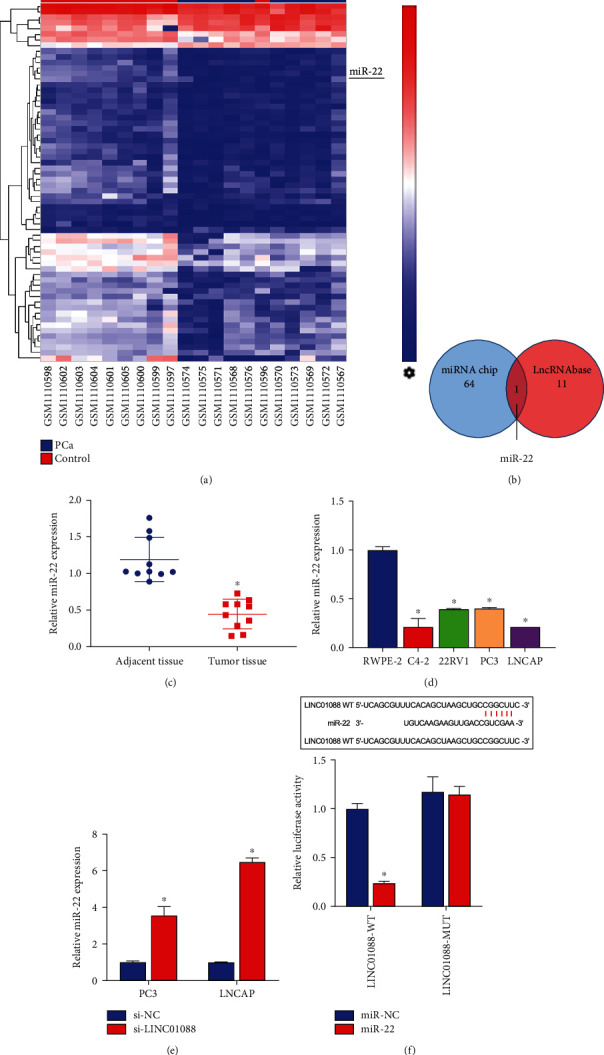
LINC01088 sponge adsorbs miR-22. (a) Hierarchical clustering heat map shows differentially expressed miRNA between the PCa and control groups. (b) The miRNA chip and the lncRNA database were combined to analyze the potential miRNAs that bind to LINC01088. (c) Relative expression of miR-22 between the PCa tumor tissues and adjacent tissues. (d) Relative expression of miR-22 in RWPE-2 and four PCa cell lines (C4-2, 22RV1, PC3, and LNCaP). (e) RT-qPCR analysis of the effect of si-LINC01088 on the expression of miR-22 in PC3 and LNCAP cells. (f) Dual-luciferase assay analysis of the binding of LINC0108 to miR-22. ^∗^*P* < 0.05.

Based on miRNA chip data analysis results, 64 differentially expressed hsa-miRNAs were identified with 5 upregulated and 59 downregulated hsa-miRNAs using 10 PCa and 10 normal prostate samples (Figure 2(a)), the joint analysis of LncBase v3 database and miRNA chip data showed that miR-22 was the only intersection among the down-regulated miRNAs (Figure 2(b)) and miR-22 was downregulated in both tissues and cells of PCa (Figures 2(c) and 2(d)). Moreover, suppressing the expression of LINC01088 increased the expression of miR-22 (Figure 2(e)). Subsequent experiments confirmed that LINC01088 directly targets miR-22 (Figure 2(f)). Here, we confirmed that LINC01088 can adsorb miR-22 to regulate PCa development.

### 3.3. CDC6 Directly Targeted miR-22

**Figure 3:**
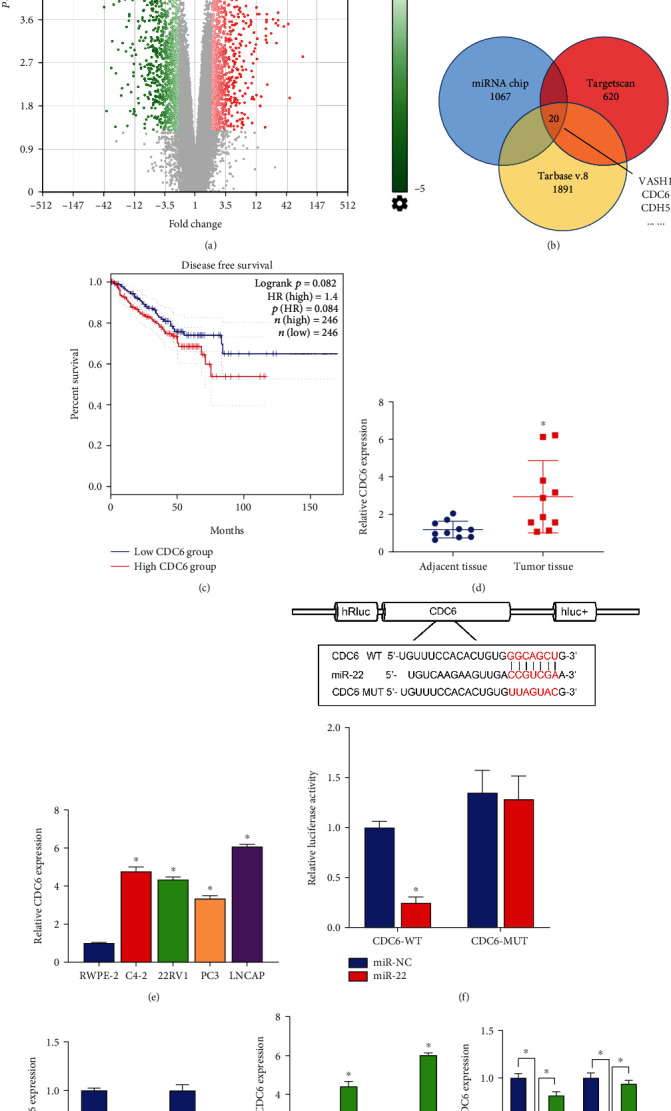
CDC6 targets miR-22 and reverses the effects of si-LINC01088 in PC3 and LNCAP cells. (a) Scatter plot shows differentially expressed mRNAs between the PCa and control groups. (b) The dual-luciferase reporter assay shows the relative luciferase activity of the CDC6-WT or CDC6-MUT plasmid between the miR-NC and miR-22 groups. (c) The disease-free survival rate was calculated in the CDC6-high or CDC6-low groups by TCGA survival analysis on the GEPIA2 database. (d) Relative expression levels of LINC01088 between the PCa tumor tissues and adjacent tissues. (e) Relative expression of LINC01088 in RWPE-2 and four PCa cell lines (C4-2, 22RV1, PC3, and LNCaP). (f) Dual-luciferase assay analysis of the binding of CDC6 to miR-22. (g) RT-qPCR analysis of the effect of si-LINC01088 on the expression of CDC6 in PC3 and LNCAP cells. (h) RT-qPCR verified the validity of overexpressing CDC6 plasmid. (i) RT-qPCR analysis of the reversal of LINC01088 expression by overexpressing CDC6 on si-LINC01088. ^∗^*P* < 0.05.

Given that miRNAs possess the capacity to regulate transcription and translation via the binding to the 3′UTR of mRNAs [33], we analyzed PCa-related differentially expressed mRNAs in the GEO dataset. The mRNA chip data analysis showed that 1,067 oncogenes were upregulated (Figure 3(a)). The combined analysis of the TargetScan database and TarBase V.8 database showed that 20 genes had overlapped, among which 2 genes had potential binding sites, namely, VASH1 and CDC6 (Figure 3(b)). A previous investigation revealed that VASH1 was downregulated in PCa [34], while the augmented expression of CDC6 conspicuously truncated the disease-free survival of PCa patients (Figure 3(c)). Therefore, we designated the CDC6 gene as a plausible target for subsequent verification. Our finding substantiated that CDC6 was highly expressed within PCa tissues and cells (Figures 3(d) and 3(e)). In addition, our dual-luciferase assay validated that CDC6 directly targeted miR-22 (Figure 3(f)). Following the suppression of LINC01088, the expression of CDC6 was downregulated within PC3 and LNCAP cells (Figure 3(g)).

### 3.4. LINC01088 Is Positively Correlated with CDC6

The association between LINC01088 and CDC6 was verified by constructing an overexpression plasmid for CDC6 (ov-CDC6). Results indicated that CDC6 were upregulated in ov-CDC6-transfected PC3 and LNCAP cells, confirming the effectiveness of CDC6 overexpressed plasmid (Figure 3(h)). In addition, after cotransfection of PC3 and LNCAP cells with ov-CDC6 and si-LINC01088, excessive CDC6 reversed the inhibition of LINC01088 expression by si-LINC01088. (Figure 3(i)). Thus, miR-22 directly targets both LINC01088 and CDC6, and LINC01088 is positively correlated with CDC6.

### 3.5. The LINC01088/miR-22/CDC6 Axis Affects PCa Development through PI3K/AKT Signaling

**Figure 4:**
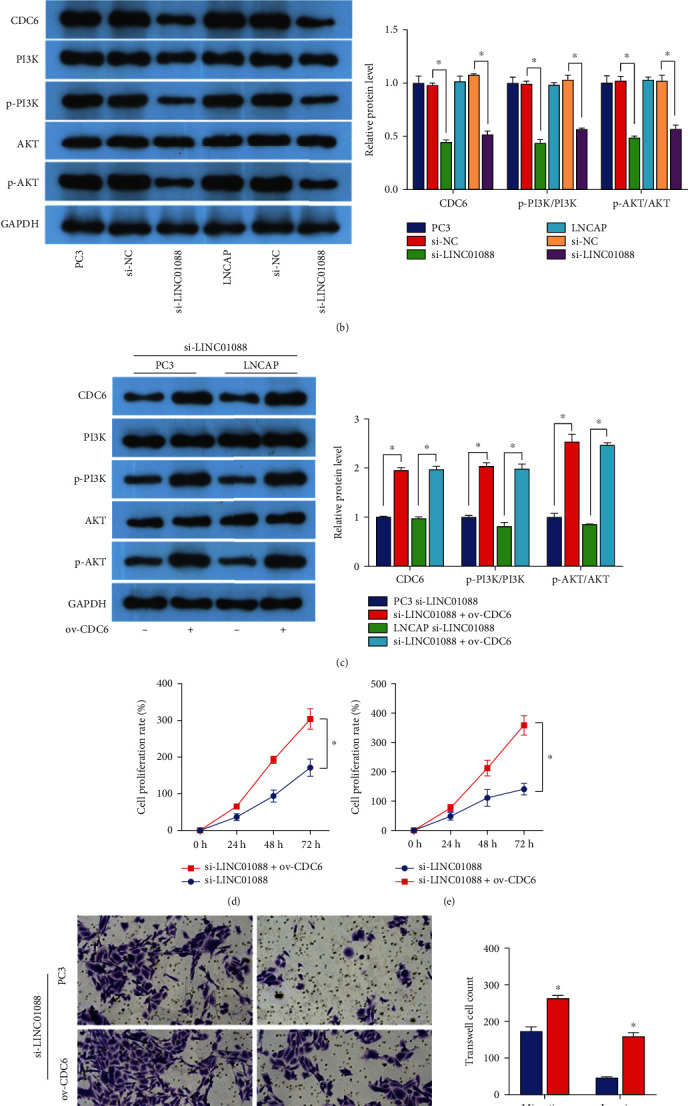
si-LINC01088 suppresses PCa cell growth via the PI3K/AKT pathway. (a) Top 10 KEGG pathway enrichment analysis. (b) Western blot analysis of si-LINC01088-mediated protein levels of CDC6, PI3K, p-PI3K, AKT, and p-AKT. (c) Western blot analysis of si-LINC01088/ov-CDC6-mediated protein levels of CDC6, PI3K, p-PI3K, AKT, and p-AKT. CCK8 analysis of the effect of si-LINC01088/ov-CDC6 co-action on the viability rate of (d) PC3 and (e) LNCAP cells. Transwell analysis of the effect of si-LINC01088/ov-CDC6 coaction on migration and invasion of (f) PC3. ^∗^*P* < 0.05.

Next, to identify the key regulatory signaling pathway, we analyzed enriched signaling pathways associated with the differentially expressed genes using the DAVID database. Among the top 10 enriched pathways, the PI3K/AKT pathway associated with 45 participating differential genes, which is the highest number of genes, was selected (Figure 4(a)). Based on Western blotting, knockdown of LINC01088 in PC3 and LNCAP cells resulted in decreased CDC6, p-PI3K, and p-AKT protein levels (Figure 4(b)). ov-CDC6 reversed the effects of LINC01088 (Figure 4(c)). Moreover, si-LINC01088 can inhibit the viability rate (Figures 4(d) and 4(e)) and migration/invasion of PC3 and LNCAP cells (Figure 4(f)). Taken together, LINC01088 affects PI3K-AKT signaling through sponge adsorption of miR-22 and regulates CDC6, thereby regulating PCa development.

## 4. Discussion

Our aim was to identify a novel lncRNA with potential therapeutic value in the treatment of PCa. Initially, we investigated the oncogenic function of LINC01088 and CDC6 and the tumor-suppressing role of miR-22 in PCa. LINC01088 and CDC6 expression were both upregulated in PCa tissues and cells, unlike miR-22, promoting PCa cell growth in vitro. We discovered that LINC01088 sponges miR-22, CDC6 is the target of miR-22, and LINC01088 is coordinately upregulated with CDC6 in PCa progression. Furthermore, LINC01088 activates the PI3K/AKT pathway.

LINC01088 is an influential regulatory factor in various cancers [8, 9]. For instance, Peng et al. discovered that LINC01088 promotes glioma progression [35], and Liu et al. observed that LINC01088 promotes proliferation and migration of non-small-cell lung cancer [8]. In a similar vein, our research showed that LINC01088 is upregulated in PCa, and its overexpression significantly reduces the disease-free survival of PCa patients. LINC01088 promotes PCa cell viability rate and migration/invasion via the PI3K/AKT signaling, indicating that LINC01088 has an oncogenic role in promoting PCa development. When LINC01088 was suppressed, the growth of PCa cells is restricted. Furthermore, LINC01088 plays a role as a ceRNA, a miRNA sponging factor, in cancers. For instance, Zang et al. found that LINC01088 inhibits the tumorigenesis of ovarian epithelial cells by sponging miR-24-1-5p [36], and Li et al. found that LINC01088 plays a ceRNA role by sponging miR-22 in the progression of colorectal cancer [9]. In our study, we demonstrated that LINC01088 sponges miR-22 and negatively regulative miR-22 in PCa. Moreover, suppressing the expression of LINC01088 promoted the expression of miR-22 in PCa cells.

miR-22 regulates the target gene to affect posttranscription or translation in PCa progression [13, 14]. Bioinformatics predicted that miR-22 targets *CDC6*, and the target mechanisms were proved by dual-luciferase reporter assay. Furthermore, CDC6 expression was negatively correlated with miR-22 expression in PCa tissues and cells. Notably, CDC6 promotes PCa progression [37, 38]. Moreover, LINC01088 is coordinately upregulated with miRNA target genes in the ceRNA axis. In our study, LINC01088 was coordinately upregulated with CDC6, si-LINC01088 reduced CDC6 expression in PCa cells, and CDC6 reversed si-LINC01088-mediated PI3K and AKT protein expression reduction in PCa cells. In addition, the slow growth of PCa cells caused by the inhibition of LINC01088 expression was significantly alleviated by the overexpression of CDC6. These results suggest that LINC01088 exerts oncogenic effects through the miR-22/CDC6 axis.

The PI3K/AKT pathway directly controls PCa [39, 40]. Shorning et al. found the PI3K-AKT signaling and PCa at the intersection of AR-MAPK-Wnt [41]. In the present study, at the time of suppression of cell viability rate, promotion of cell apoptosis, and suppression of migration/invasion, PI3K/AKT phosphorylation levels were reduced. LINC01088, miR-22, and CDC6 control the PI3K/AKT pathways to regulate PCa progression. si-LINC01088 or miR-22 suppressed PCa cell growth and decreased PI3K/AKT phosphorylation levels. CDC6 promoted cancer cell growth and enhanced PI3K/AKT signaling and reversed the effect of si-LINC01088 on PCa cells.

Interlaboratory variability may also affect the development of therapeutic strategies targeting the LINC01088/miR-22/CDC6 axis. Despite the potential limitations, this study provides a foundation for further research on the LINC01088/miR-22/CDC6 axis as a potential therapeutic target for PCa treatment. Future studies can build upon these findings and investigate the molecular mechanisms underlying the regulatory interactions among LINC01088, miR-22, and CDC6 in PCa. Therefore, it is important to conduct validation studies to confirm the therapeutic potential of this axis across multiple laboratories and patient populations before initiating clinical trials.

In conclusion, this study confirms the LINC01088/miR-22/CDC6 axis as a potential therapeutic target, providing more directions and theoretical basis for the treatment of PCa.

## Data Availability

The authors declare that all other data supporting our findings are available within the article and from the corresponding authors upon reasonable request.
